# Age at gonadectomy and risk of overweight/obesity and orthopedic injury in a cohort of Golden Retrievers

**DOI:** 10.1371/journal.pone.0209131

**Published:** 2019-07-17

**Authors:** Melissa Simpson, Sharon Albright, Barbara Wolfe, Erin Searfoss, Katie Street, Kelly Diehl, Rodney Page

**Affiliations:** 1 Morris Animal Foundation, Denver, Colorado, United States of America; 2 American Kennel Club Canine Health Foundation, INC, Raleigh, North Carolina, United States of America; 3 Flint Animal Cancer Center, College of Veterinary Medicine and Biomedical Sciences, Colorado State University, Fort Collins, Colorado, United States of America; McMaster University, CANADA

## Abstract

**Introduction:**

In the United States, gonadectomy is common and widely promoted as a component of responsible pet ownership. The recent publication of several studies examining the effect of gonadectomy on future health has challenged long-held assumptions and recommendations for gonadectomy in companion animals. The purpose of this study was to characterize the associations between gonadectomy and two outcomes: overweight/obesity and orthopedic injuries, in a large prospective study of Golden Retrievers.

**Methods:**

Age at gonadectomy was divided into four categories: intact (reference), ≤ 6 months, > 6 months ‒ ≤ 12 months, and > 12 months. Dogs with a Purina Body Condition Score of 7 or greater were classified as overweight or obese. Orthopedic injuries considered were the first instance of veterinary-reported cranial cruciate ligament injury and clinically evident osteoarthritis. We performed survival analyses on a cohort of Golden Retrievers to estimate the associations of interest using proportional hazards. We adjusted for age at study enrollment, owner-reported activity level, and dog’s sex.

**Results:**

Compared to intact dogs, all gonadectomy age categories showed increased risk for the development of overweight/obesity. (≤ 6 months, HR: 1.81, 95% CI: 1.36–2.40), p-value: <0.0001; 6 months to ≤ 12 months, HR: 2.21, 95% CI: 1.77–2.73, p-value: < 0.0001; > 12 months, HR: 1.56, 95% CI: 1.24–1.96, p-value: 0.0001). Compared to intact dogs, dogs who were ≤ 6 months at gonadectomy had increased risk for orthopedic injury (HR: 4.06, 95% CI: 2.15–7.67, p-value: <0.00001).

**Discussion:**

This study presents prospectively acquired data demonstrating that gonadectomy is a risk factor for both overweight/obesity and chronic non-traumatic orthopedic injuries in a prospective cohort of Golden Retrievers. Our data suggest that gonadectomy at any age is a risk factor for overweight or obesity, but delaying gonadectomy until dogs are at least 6–12 months of age may help to decrease the risk for orthopedic injury.

## Introduction

In the United States, gonadectomy is common and widely promoted as a component of responsible pet ownership. However, as veterinary medicine continues to embrace evidence-based recommendations, gonadectomy in pets is receiving increased attention. Dog owners and veterinarians are questioning how to balance the potential adverse health events and benefits associated with reproductive hormone dose changes that result from gonadectomy. Specifically, questions of timing of gonadectomy to optimize positive outcomes while minimizing negative ones remain unanswered. Adverse health outcomes associated with gonadectomy in dogs include urinary incontinence, [1, 2] obesity, [3, 4] orthopedic injuries, [5] and cancer.[6, 7] In addition to health outcomes, studies have examined cause of mortality and age at mortality based on reproductive status. Gonadectomized dogs have longer life spans [8, 9] but cancer appears to be a more common cause of death in gonadectomized dogs. [8] However, studies focused on these outcomes offer conflicting results.

The incidence of overweight and obesity in pet dogs has steadily increased in recent years.[10] Overweight and obesity are associated with numerous chronic morbidities including disorders of the musculoskeletal, endocrine, and cardiovascular systems as well as potentially implicated as a risk factor for cancer.[11, 12] Because of the deleterious outcomes associated with overweight/obesity, there is increased attention to the causes and prevention of this condition.

Several studies have linked gonadectomy with increased risk for overweight or obesity. A large study of medical records from a national network of veterinary hospitals found gonadectomized dogs were at increased risk for overweight, but the age at which gonadectomy occurred was not associated with that risk.[4] In addition, three years after gonadectomy, the risk for overweight in intact and gonadectomized dogs converged. A similar study in the United Kingdom found that spayed females were at increased risk of obesity compared to intact males but neutered males and intact females did not differ in their risk compared to intact males.[3] Another report of dogs adopted from a shelter found that younger age at gonadectomy was associated with decreased obesity compared to dogs who were older at the time of gonadectomy, but this study did not include an intact comparison group.[13]

In addition to lingering issues regarding the association between obesity/overweight and gonadectomy, similar questions have been raised regarding the lifetime exposure-dose of reproductive hormones and the development of orthopedic disease. Mechanistic evidence suggests that reproductive hormones initiate growth plate closure in long bones and that these hormones are responsible for maintaining tendon, ligament, and muscle integrity.[14, 15] Abrupt removal of these hormones may lead to weakening of joints and musculature as well as changes in joint conformation.[16, 17]

The literature regarding the influence of hormone removal and cruciate ligament injury in dogs is conflicting. Several studies have documented associations between cruciate ligament injuries (as well as other adverse health events) and gonadectomy in both young and old dogs.[5, 18–21] In contrast, two studies found no difference between intact and gonadectomized dogs.[22, 23] These studies were case-control and therefore subject to potential misclassification of exposure timing. The study described herein avoids potential exposure misclassification since gonadectomy and outcome data are collected in real time. The authors are not aware of a prospective study evaluating the timing of gonadectomy and the risk of orthopedic injury.

Elucidating the association between timing of gonadectomy, overweight/obesity, and orthopedic injury, as well as understanding how overweight/obesity affects the association between gonadectomy and orthopedic injury, may help veterinarians and owners make informed decisions about if and when to perform gonadectomy.

The purpose of this study was to characterize the associations between gonadectomy and two outcomes: overweight/obesity and orthopedic injuries in a large, lifetime prospective cohort of Golden Retrievers in the United States. In addition, the study investigated the degree to which the association between gonadectomy and orthopedic injuries can be mitigated by maintaining a healthy body weight using a mediator analysis.

## Methods

### Study population

This study was performed using data collected as part of the Golden Retriever Lifetime Study. Details about study design and cohort characteristics have been published. [24, 25] Briefly, the Golden Retriever Lifetime Study is a prospective study of privately cared-for Golden Retrievers living throughout the contiguous 48 United States. The goal of the study is to identify genetic, environmental, nutritional, and lifestyle risk factors for cancer. Because dogs were young (≥ 8 weeks and < 2 years of age) at the time of enrollment, it is a robust study design for examining numerous health outcomes.

**Fig 1:**
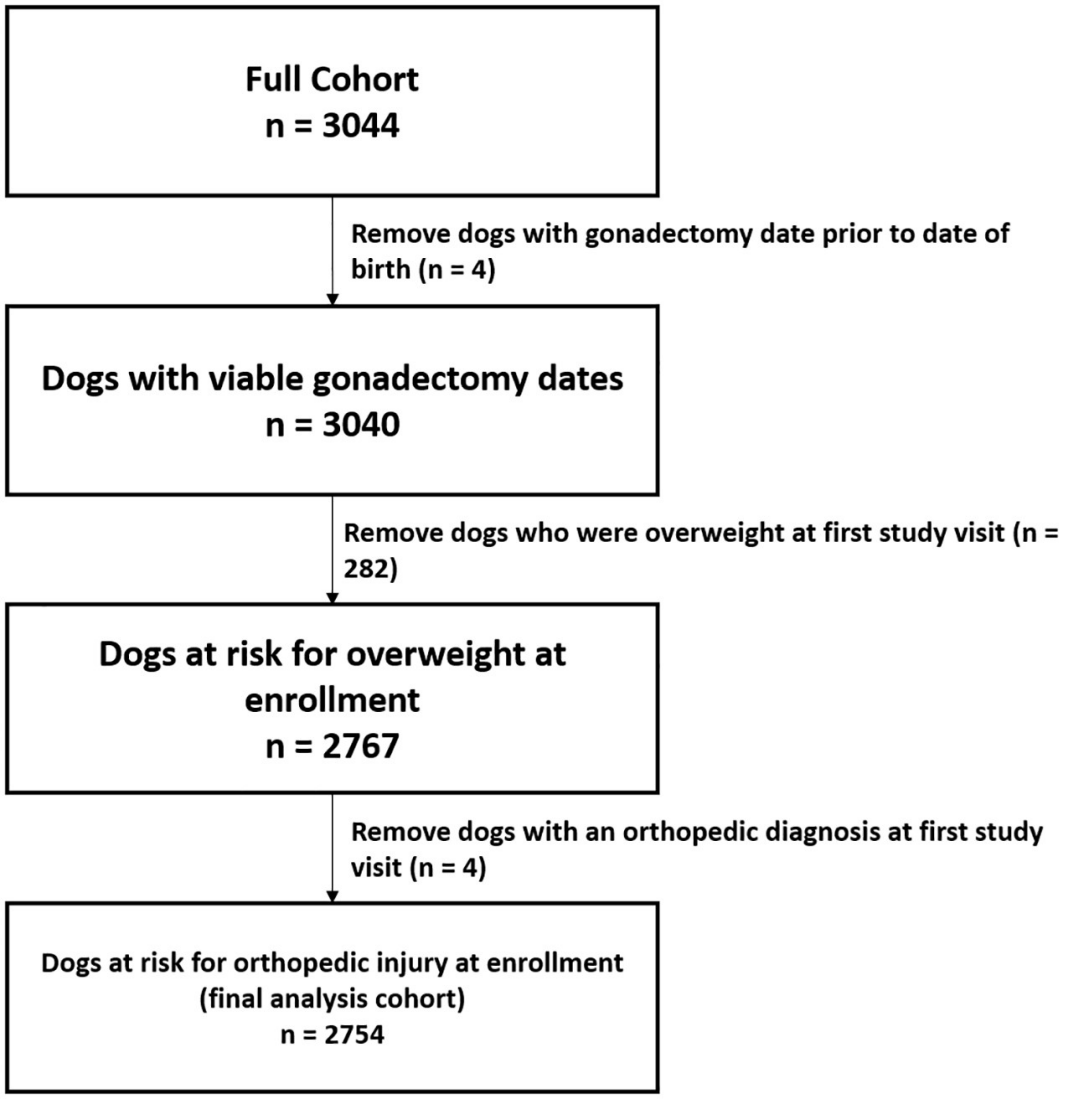
Summary of the analysis cohort used in the study of the association between age at gonadectomy and the risk for non-traumatic orthopedic injuries in the Golden Retriever Lifetime Study.

Details about the portion of the cohort included in this study are highlighted in Fig 1. Dogs were excluded from this analysis if they were overweight or obese at the time of study enrollment and/or they had a pre-existing diagnosis of orthopedic injury (i.e. left-censored). The final analysis cohort for this study was 2,764 dogs (91% of the full cohort) with 11,214 unique veterinary visits (minimum visits: 1, maximum visits: 7, mean visits per dog: 4).

### Exposure of interest

Gonadectomy, as reported by dog owners, is the main exposure of interest. Owners were asked to report their dogs’ reproductive statuses annually until they are no longer intact. When they report that their dog underwent gonadectomy, they are asked to report the month and year that the procedure took place. In cases where answers did not make logical sense (Eg: the date of gonadectomy preceded date of birth), owners and veterinarians were contacted to verify the actual date. All but four nonsensical dates were rectified.

To calculate the age at gonadectomy, we used the 15^th^ day of the month and year from owner reported information and subtracted from the date of birth. To compare age at gonadectomy, we used the following categories: ≤ 6 months of age at gonadectomy, >6 to ≤ 12 months, > 12 months, and intact (reference group). We chose these categories to approximate different phases of sexual maturation at the time of gonadectomy: ≤ 6 months contains dogs who did not experience pubertal changes, > 6 months to ≤ 12 months contains dogs who were in puberty at gonadectomy and > 12 months contains dogs who were sexually mature at the time of gonadectomy. [21]

### Outcomes of interest

We have two outcomes of interest in this study. The first was overweight or obesity. We assessed this outcome using veterinarian-reported Purina Body Condition Score (BCS). Dogs were classified as overweight or obese if they had a BCS greater than 6/9 because these designations are agreed upon and validated definitions for overweight/obesity using this published scoring system. [26] The second outcome of interest is the first instance of veterinary-reported clinically evident osteoarthritis or cranial cruciate ligament injury. Veterinarians reported these diagnoses on an annual questionnaire and used medical records to report the date of diagnosis.

### Statistical analysis

To investigate the association between overweight/obesity and timing of gonadectomy, we specified a Cox proportional hazards model with a common origin defined as enrollment in the Golden Retriever Lifetime Study. [27, 28] We allowed for repeated instances of overweight/obesity within an individual by specifying a start and stop time for each observation interval and using the counting process. Gonadectomy status could vary over time in this model. For example, a dog was enrolled in the study at 2 months of age and underwent gonadectomy at 35 months of age and has completed study visits at the following ages: 2 months, 14 months, and 26 months of age. Thus, this dog’s gonadectomy status would be recorded as intact, intact, >1 year at gonadectomy for each respective study visit. We adjusted for sex, age at enrollment, and owner-reported dog’s physical activity.

To investigate the association between orthopedic injuries and timing of gonadectomy, we used a Cox proportional hazards model with the first veterinary-reported instance of one of the orthopedic injuries of interest as the outcome. Once a dog received one of these diagnoses, they were removed from the risk pool (i.e. we did not allow repeated events). The adjusted model in this analysis included sex, age at enrollment, owner-reported dog’s physical activity (collected annually as: none, little, moderate, or very active; we collapsed none and little into the ‘sedentary’ category), and overweight/obesity. Gonadectomy status, overweight/obesity, and physical activity could vary over time. All descriptive characteristics contained in the tables are from a dog’s most recent visit, even for those characteristics that can vary over time in the statistical models.

To test whether the gonadectomy-timing categories differed from each other, we used three linear contrasts for the overweight/obesity and orthopedic injury outcomes. The contrasts performed were ≤ 6 months vs. >6months to ≤12 months, ≤ 6 months vs. > 12 months, and >6months to ≤12 months vs. > 12 months. We also tested to see if the associations between gonadectomy and the outcomes of interest differed by sex by testing the interaction of gonadectomy status and sex. We were unable to use the gonadectomy-age categories for this test because the data were sparse, so we used gonadectomy as a binomial variable.

When determining whether an association between gonadectomy and orthopedic injury was mediated by overweight/obesity, the parameter estimates generated from the models specified above were used to estimate the direct and indirect effects of gonadectomy on orthopedic injury. P-values for mediation were generated using the Sobel method. [29] To estimate the population attributable fraction (PAF) of cases of orthopedic injury attributable to gonadectomy ≤ 6 months and overweight/obese, we used methods outlined by Mason *et al*. [30] This method allows for adjustment for mediating factors.

All analyses were performed using Statistical Analysis Software v9.4 (SAS, Cary NC). The data used for all analyses can be found in the S1 Dataset.

## Results

**Fig 2:**
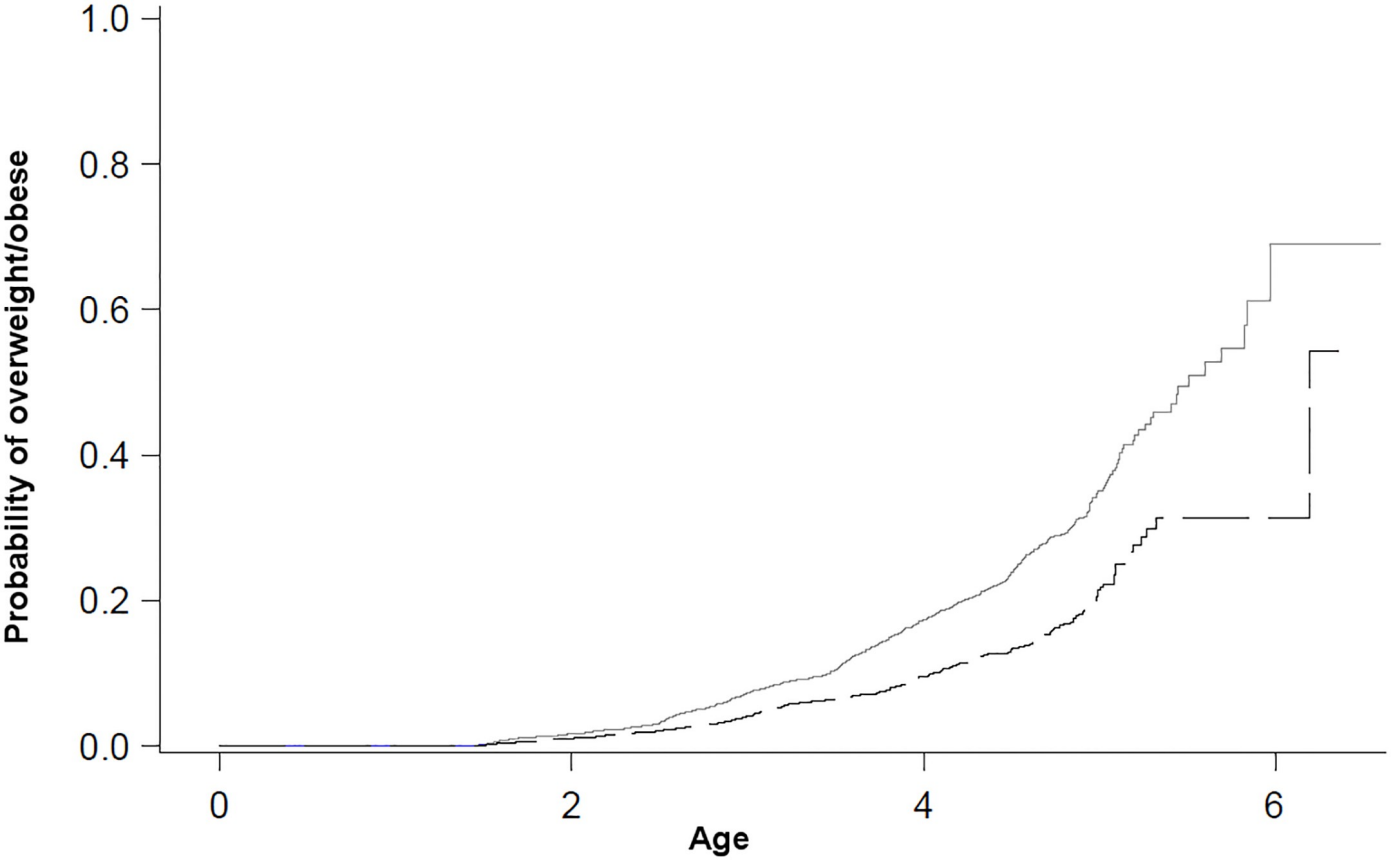
Kaplan-Meier failure plot for overweight/obesity as a function of age stratified by gonadectomy status in a cohort of Golden Retrievers. Intact = dashed black line, gonadectomized = solid grey line.

Fig 2 shows the Kaplan-Meier survival curves for overweight/obesity as a function of age and stratified by gonadectomy status. The solid gray line represents gonadectomized dogs and shows that the probability for overweight/obesity is higher for this group. Furthermore, Fig 2 demonstrates that the increased probability persists as of middle-age (i.e. the probability for overweight between the two groups does not converge).

**Fig 3:**
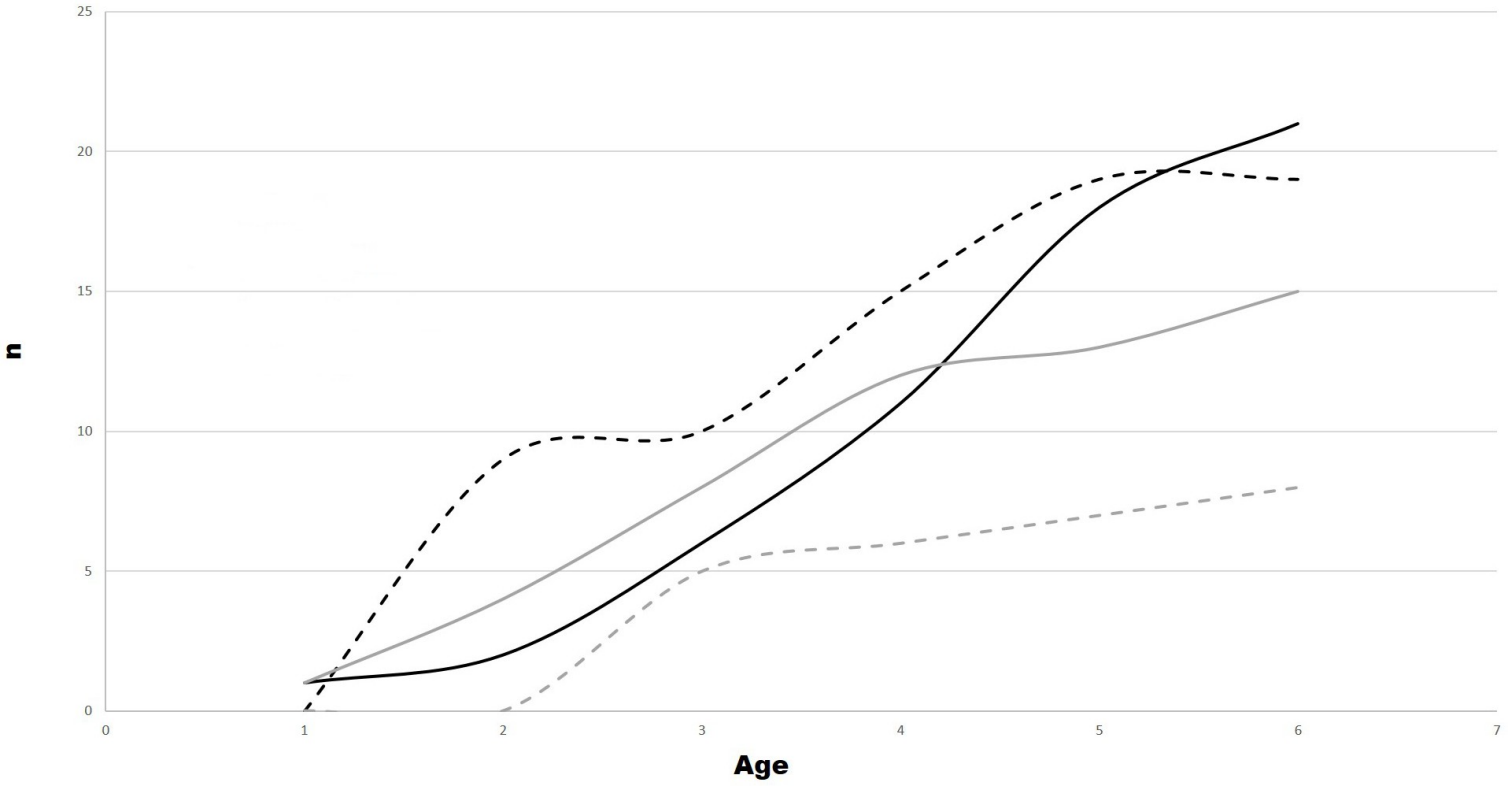
Cumulative counts of orthopedic injury cases by age and gonadectomy category. The solid black line is intact dogs, the dashed black line shows dogs gonadectomized ≤ 6 months, the solid gray line shows dogs gonadectomized 6 months– 1 year, and the dashed gray line show dogs gonadectomized > 1 year of age.

The cumulative count of orthopedic injury cases based on dogs’ age and stratified by gonadectomy category is presented in Fig 3.

Table 1 details the descriptive characteristics, unadjusted, and adjusted hazard ratios for the risk for overweight/obesity. In both unadjusted and adjusted analyses, compared to intact dogs, all gonadectomy age categories experienced an increased risk for the development of overweight/obesity.

**Table 1:** Descriptive characteristics, unadjusted, and adjusted hazard ratios for the risk for overweight/obesity in a cohort of Golden Retrievers as a function of gonadectomy.

Characteristic	Overweight or obesity					p-value
	Yes	No	unadjusted HR (95% CI)	p-value	adjusted HR (95% CI)	
	n = 639 (23%)	n = 2113 (77%)				
Gonadectomy age n(%)						
Intact	186 (23)	1060 (50)	ref		ref	
≤6 months	88(14)	185 (9)	1.95 (1.48–2.56)	<0.0001	1.81 (1.36–2.40	<0.0001
6 months—1 year	187 (29)	390 (18)	2.39 (1.92–2.97)	<0.0001	2.21 (1.77–2.73)	<0.0001
> 1 year	180 (28)	478 (23)	1.72 (1.36–2.40)	<0.0001	1.56 (1.24–1.96)	0.0001
Age at enrollment in months—mean(sd)	4.1(3.7)	4.2(3.7)	0.31 (0.23–0.41)	<0.0001	0.30 (0.22–0.41)	<0.0001
Female n(%)	316 (49)	1062 (50)	1.08 (0.91–1.28)	0.38	1.11 (0.93–1.77)	0.23
Activity level n(%)						
very active	88(14)	642 (30)	ref		ref	
moderately active	484 (76)	1382 (65)	1.52 (1.28–1.81)	<0.0001	1.48 (1.23–1.77)	<0.0001
Sedentary	68 (11)	88(4)	2.42 (1.75–3.33)	<0.0001	2.22 (1.59–3.09)	<0.0001

When comparing the gonadectomy age groups to one another, the dogs who were gonadectomized younger than six months did not differ from the 6–12 month or older than 12 month gonadectomy groups (HR: 0.82, 95% CI: 0.66–1.01, p = 0.06; HR: 1.16, 95% CI: 0.93–1.44, p = 0.19, respectively). However, the dogs who were gonadectomized from 6–12 months had a 42% increased risk for overweight/obesity compared to the group that was older than 12 months at the time of gonadectomy (HR: 1.42, 95% CI: 1.19–1.69, p = 0.0001).

Finally, we tested whether the association between gonadectomy and overweight/obesity differed by sex and found a significant interaction term (p = 0.0006). Stratified analyses showed that among intact dogs, females are at 43% increased risk for overweight/obesity compared to males (HR: 1.43, 95% CI: 1.14–1.81). Among gonadectomized dogs, females had a 24% lower risk for overweight/obesity (HR: 0.76, 95% CI: 0.65–0.88).

We had 41 veterinary-reported cruciate ligament injuries and 22 reports of clinically evident osteoarthritis. Table 2 shows descriptive characteristics, unadjusted, and adjusted hazard ratios for the risk for orthopedic injury. Dogs who were 6 months or younger at gonadectomy had more than 300% increased risk for orthopedic injuries compared to intact dogs (Table 2). In addition, overweight and obesity almost doubled the risk for orthopedic injuries (Table 2).

**Table 2:** Descriptive characteristics, unadjusted, and adjusted hazard ratios for the risk for orthopedic injury in a cohort of Golden Retrievers as a function of gonadectomy.

characteristic	orthopedic injury					p-value
	No	Yes	unadjusted HR (95% CI)	p-value	adjusted HR (95% CI)	
	n = 2691 (85%)	n = 63 (2%)				
gonadectomy status n(%)						
intact	1224 (45)	22 (35)	ref		ref	
≤6 months	254 (9)	19 (11)	4.04 (2.18–7.48)	0.0001	4.06 (2.15–7.67)	<0.0001
6 months—12 months	562 (21)	15 (24)	1.59 (0.83–3.06)	0.17	1.62 (0.83–3.18)	0.16
older than 12 months	651 (24)	7 (30)	0.61 (0.27–1.36)	0.23	0.64 (0.28–1.44)	0.28
overweight/obese n(%)	612 (23)	29 (46)	2.05 (1.14–3.69)	0.02	1.97 (1.07–3.62)	0.03
age at enrollment in months mean(sd)	4.1 (3.7)	5.0 (4.6)	1.35 (0.65–2.80)	0.41	1.56 (0.75–3.24)	0.23
female n(%)	1353 (50)	25 (40)	0.66 (0.4–1.09)	0.10	0.73 (0.44–1.22)	0.23
activity level n(%)						
very active	713 (27)	17 (27)	ref		ref	
moderately active	1822 (68)	44 (70)	0.58 (0.33–1.01)	0.05	0.47 (0.27–0.81)	0.007
sedentary	154 (6)	2 (3)	1.21 (0.48–3.01)	0.69	0.88 (0.35–2.23)	0.79

In the adjusted model, we compared gonadectomy age groups to one another and found that all groups differed from each other (Table 3). In all comparisons, younger age at gonadectomy was associated with increased risk for orthopedic injury.

The association between gonadectomy and orthopedic injuries did not differ by sex (p = 0.23).

**Table 3:** Comparison of gonadectomy age groups and the risk for orthopedic injuries in a cohort of Golden Retrievers.

Comparison	HR	95% CI	p-value
≤ 6 months vs. 6 to ≤12 months	2.24	(1.25–4.93)	0.008
6 to ≤12 months vs. > 12 months	2.56	(1.08–2.25)	0.03
≤ 6 months vs. > 12 months	6.36	2.74–14.77)	<0.0001

When testing how much of the association between gonadectomy and orthopedic injuries is mediated by overweight/obesity, we found that 16% of the total association was mediated by overweight/obesity. This mediation was not significant (p = 0.07). Since only the dogs who were ≤ 6 months at gonadectomy had increased risk for orthopedic injury, we performed the mediation analysis comparing this group to all other dogs and found that 7% of the association was mediated by overweight/obesity (p = 0.05).

The PAF for orthopedic injuries among dogs gonadectomized at ≤ 6 months compared to all other groups was 20% and the total PAF for both overweight/obese and gonadectomy at ≤ 6 months was 48%.

## Discussion

This study presents data showing that gonadectomy is a risk factor for both overweight/obesity and non-traumatic orthopedic injuries in a prospective cohort of Golden Retrievers. These data suggest that the risk for orthopedic injuries associated with gonadectomy may be somewhat mitigated by maintaining dogs at a healthy body condition, but that the majority of the association is related to gonadectomy. Our findings confirm similar results published by other investigators [3–5, 21] and extends these findings to examine the length of time during which reproductive hormones may be acting on the development of overweight/obesity, and to include evaluation of reproductive hormone status on chronic orthopedic injuries.

Our finding that, although all gonadectomized dogs are at increased risk for overweight/obesity compared to intact dogs, there is not a clear linear association when comparing different gonadectomy age groups. This suggests that gonadectomy is a risk for overweight/obesity, but the timing may not be important. As our cohort matures, it will be important to examine if the association between gonadectomy and overweight is attenuated by age or if the association persists into late life. In addition, it will be important to assess whether factors such as the length of time that dogs are exposed to the pro-inflammatory state of obesity or if a time during a dog’s development wherein the adverse effects of overweight are amplified (i.e. a sensitive period effect), increase the risk for chronic adverse health outcomes.

An important detail to consider is the evidence from human medical research that obesity confers non-linear increased risks for adverse health events such as cancer. [31] While the distinction between overweight and obesity may also be important in veterinary patients, we were unable to study this question in this paper because of the potential for misclassification of obesity and small numbers in the highest BCS range.

Important differences exist between this cohort and a general population of dogs seen in private practice besides the fact that they are exclusively pure bred golden retrievers. Two studies that used more generalized populations of dogs report rates of overweight/obesity of approximately 53–59% [3, 4] compared to 23% in this cohort. Owner-driven decisions associated with a higher standard of health care such as vaccine uptake and obtaining pet health insurance are higher in our cohort compared to the population of dogs seen in general practice. [24, 25] While these differences may limit the generalizability of our findings, the fact that what we found is consistent with other studies suggests that the results are robust across populations of dogs. These differences limit the generalizability of the PAF that we calculated.

Although only the dogs who were ≤ 6 months of age when gonadectomized were at increased risk for orthopedic injury compared to intact dogs, all three age groups differed from each other. This finding suggests the potential for a dose-response relationship between the amount of time that a dog is exposed to reproductive hormones and the risk for orthopedic injury. Future studies may want to examine biomarker expression associated with gonadectomy and the risk for chronic health outcomes, including but not limited to orthopedic injuries.

The significant interaction term for sex by gonadectomy showing females have different risk for overweight/obesity compared to males depending on whether they were gonadectomized (p-value for interaction = 0.0006) is comparable to the data published by Lefevre *et al*. In that study of different dog breeds, sexually intact males were at decreased risk for overweight or obesity compared to females. However, there was no difference between sexes among gonadectomized dogs. [4] One potential explanatory mechanism is that the presence of male reproductive hormones confers greater protection against fat accretion than female reproductive hormones, but when these hormones are removed other factors can play a larger role in the development of overweight.

Although standardized and commonly used by veterinarians, body condition score is a subjective assessment which introduces the potential for misclassification of one of our outcomes. However, there is published evidence that body condition score is repeatable, even when assessed by different veterinarians [26, 32] and, in our data, we found that lower owner-reported physical activity was associated with a higher BCS, which suggests that veterinary assessment of BCS is reliable in this cohort (Table 1).

Obesity and orthopedic injury are complex and multifactorial in etiology. Nutrition and genetics are potentially important contributing factors to the associations that we found in this study. The structure of our study including a thorough dietary database for each dog and collection of biological samples including DNA, serum, urine, hair, nails and feces will permit future evaluation of multi-factorial interactions. In addition, because Golden Retrievers represent a homogeneous population and share many of the same environmental pressures as the people who own them, they may be a useful surrogate to identify potential candidate genes for obesity and other conditions in humans.

Recommendations regarding the timing of gonadectomy should take many factors into account, including breed and dog size. In addition, fears about negative health outcomes associated with intact status, such as the development of mammary cancer, have informed gonadectomy recommendations independent of dog size and breed. The data presented here suggest that, at least in golden retrievers, optimal timing of gonadectomy would be older than one year of age to avoid orthopedic disease but gonadectomy at any age is a risk factor for overweight and obesity. However, we have not yet estimated the association between gonadectomy and other health outcomes and veterinarians need to consider the individual needs of their patients and clients when advising on health decisions.

## Supporting information

S1 Dataset The data used for all analyses contained in this manuscript can be found here. (SAS7BDAT)
